# Impact of Nonsurgical Periodontal Treatment on Blood Pressure: A Prospective Cohort Study

**DOI:** 10.1055/s-0043-1772246

**Published:** 2023-09-20

**Authors:** Neus Lanau, Javier Mareque-Bueno, Michel Zabalza

**Affiliations:** 1 Department of Oral Medicine and Public Health, Faculty of Dentistry, Universitat Internacional de Catalunya, Barcelona, Spain; 2 Department of Oral Medicine and Public Health, Faculty of Dentistry and Faculty of Medicine, Universitat Internacional de Catalunya, Barcelona, Spain

**Keywords:** periodontitis, hypertension, prehypertension, blood pressure

## Abstract

**Objectives** Arterial hypertension and periodontitis are two of the most common diseases worldwide and recent evidence supports a causal relationship between them. Despite all antihypertensive strategies, an important number of patients are undiagnosed and a large number of the diagnosed fail to achieve optimal blood pressure (BP) measurements. Some studies point out that periodontal treatment could have positive effects on BP levels. The aim of this study is to determine if nonsurgical periodontal treatment can help BP level control in prehypertensive patients with periodontitis.

**Materials and Methods** Thirty-five patients were included in the study and received nonsurgical periodontal treatment according to necessity. Clinical data, periodontal data, and BP measurements were taken at baseline, periodontal re-evaluation visit (4–6 weeks after treatment), and 6-month follow-up.

**Results** Periodontal treatment caused a statistically significant reduction ( *p* < 0.05) of systolic blood pressure (SBP) and diastolic blood pressure (DBP) at re-evaluation visit of 4.7 ( *p* = 0.016) and 3.4 mm Hg ( *p* = 0.015), respectively. The effect was maintained at 6-month follow-up visit with a reduction in SBP and DBP of 5.2 ( *p* = 0.007) and 3.7 ( *p* = 0.003) mm Hg, respectively.

**Conclusion** Despite the limitations of this study, it suggests that nonsurgical periodontal treatment can be effective in lowering BP levels in patients with prehypertension and periodontitis. Moreover, it highlights the importance of dentists in prevention, detection, and control of this important cardiovascular risk factor.

## Introduction

Arterial hypertension is one of the most prevalent cardiovascular risk factors. 1 It is estimated to affect 1.4 billion people (>30% of world population) and cause around 10 million deaths per year worldwide, representing the first cause of premature death. 2 In this context, early detection of hypertensive patients and their medical control is essential in order to prevent cardiovascular events.

In addition, and despite all the antihypertensive strategies, it is estimated that 15 to 50% of people are undiagnosed 3 and around 70% of the diagnosed patients fail to achieve optimal blood pressure (BP) measurements. 4

The concept of prehypertension or high normal pressure encompasses patients who have systolic blood pressure (SBP) values between 130 and 139 mm Hg and/or diastolic blood pressure (DBP) values between 80 and 89 mm Hg. ^2^ This condition is associated with three more times probabilities of developing hypertension. 1 Moreover, it has been linked with worse cardiovascular risk profile, higher prevalence of metabolic disorders, and cardiovascular mortality. 5 6 7

Periodontal diseases, gingivitis and periodontitis, are one of the most prevalent chronic multifactorial inflammatory diseases worldwide. 8 It is estimated that 20 to 50% of the population is affected. 1 8 Periodontal diseases are caused by bacteria and not only affect support tissues around teeth but can also cause endothelial dysfunction, metabolic dysregulation, and systemic inflammation. 9 Likewise observational and experimental evidence suggests the importance of systemic inflammation in the development and progression of hypertension. 3

Recent evidence supports a causal relationship between hypertension and periodontitis. 9 10 Regarding this relationship, it is important to take into account, for example, the genetic predisposition of both diseases 11 12 and the effect that local inflammation of periodontal tissues has into systemic inflammation and vascular endothelium. 11 13 14 Patients with moderate or severe periodontitis tend to present higher BP measurements and have 30 to 70% more probability to develop hypertension 10 13 15 . In some recent medical guides, 16 periodontitis is now being considered as a cardiovascular risk factor. Moreover, some studies conclude that periodontal treatment helps reducing BP measurements. 16 17 18 19 20 21 22 23

Therefore, with this interventional prospective cohort study, we aimed to investigate if nonsurgical periodontal treatment could have a positive effect in BP levels, in patients with periodontitis and prehypertension.

## Materials and Methods

The participants in our study were consecutive patients from general dental practice in Barcelona, Spain, enrolled between January 2021 and March 2022. This study was approved by the Ethics Committee of Hospital Universitario Dexeus Grupo Quironsalud with code 2018/ODI-2018-01. Written consent to participate in the study was given by all patients.

Adult patients aged 18 or older, diagnosed with prehypertension, were enrolled into the study if they also presented with moderate-to-severe periodontitis. Moreover, patients must have 10 or more teeth and full capacity to understand, authorize, and sign informed consent. Exclusion criteria included acute and major chronic inflammatory/immune disorders, chronic diseases, and malignancies (within the last 5 years) as assessed by the examining clinician. Patients who had received treatment with medications known to affect periodontal status and patients using systemic or local immunosuppression within the previous 6 months were excluded, as were patients with any cause of secondary hypertension; meaning patients with hypertension caused by medications, alcohol, or drug consumption or hypertension caused by other systemic, metabolic, or immune diseases such as hyperthyroidism. Moreover, patients with necrotizing periodontitis, patients who had periodontal treatment in the previous 6 months and breastfeeding, and pregnant women were also excluded.

### Definition of Periodontitis

Regarding the Classification of Periodontal and Peri-implant Diseases and Conditions 24 established in 2017 in the American Academy of Periodontology and the European Federation of Periodontology World Workshop, a patient is considered a “periodontitis case” in the context of clinical care if:

- Interdental CAL (clinical attachment loss) is detectable at two or more adjacent teeth.
- Presence of buccal or oral CAL more than or equal to 3mm with pocketing more than or equal to 3mm detectable at 2 or more adjacent teeth (CAL only caused by periodontitis causes).

Moreover, each periodontitis case can be classified regarding stage (severity of periodontitis, from I to IV) and grade (expected progression, biological characteristics and risk factors). The stage takes into account the severity (CAL, radiographic bone loss and tooth loss), complexity (probing pocket depth [PPD] and type of bone loss horizontal or vertical), and extent and distribution. Regarding grade, a moderate progression is always assumed and evidence must be found to change grade into slow or rapid rate of progression. 24

### Definition of Hypertension and Prehypertension

According to the 2018 European Society of Cardiology (ESC) guidelines, 2 optimal BP values are defined as SBP less than 120 mm Hg and DBP less than 80 mm Hg. Hypertension is defined as SBP more than or equal to 140 mm Hg and/or DBP more than or equal to 90 mm Hg diagnosed in a medical office. High-normal BP (formerly prehypertension) is defined as SBP 130 to 139 mm Hg and/or DBP more than or equal to 85 to 89 mmHg. ^2^ These patients were the target of our study.

### Sample Size

A calculated minimum of 37 patients were necessary to recognize a statistically significant difference of 5 mm Hg in BP between visits, accepting an alpha risk of 0.05 and a beta risk of 0.2 in a two-sided test. The standard deviation was assumed to be 10 and a drop-out rate of 15% was anticipated. A final sample size of 35 patients was enrolled.

### Study Dynamics and Patient Information

This study consists of three visits: baseline, periodontal re-evaluation (4-6 weeks after the treatment), and follow-up at 6 months. During all the study, STROBE statement (Strengthening the Reporting of Observational Studies in Epidemiology) 25 was followed.

The data collected were the following:

a) *Sociodemographic data* : age, sex, ethnicity, height, weight, and body mass index (BMI).
b) Periodontal data:
Dental habits questionnaire: frequency and type of brushing, type of interdental hygiene (interproximal brushes, dental floss, or none) and frequency, presence of bleeding while brushing, and regularity of dental appointments ( Supplementary Table S1 , available in the online version>).
  - Electronic periodontogram recording PPD, CAL, and bleeding on probing (BOP). Third molars if present were excluded of the data analysis.
c) Clinical data:
  - Cardiovascular risk factors: diabetes mellitus, dyslipidemia, systemic diseases, and family background.
  - Toxic habits: smoking (number of cigarettes per day), alcohol consumption (number of drinks per day/week).
- Healthy habits: healthy diet, consumption of carbonated, and sugared drinks, physical activity ( Supplementary Table S2 , available in the online version>).

**Table 1:** Baseline characteristics of the participants in the study

	Total participants ( *n* = 35)	Male ( *n* = 18) 51.4%	Female ( *n* = 17) 48.6%
Age, mean (years)	45.42 (28–65)	45.42 (30–65)	45.42 (28–58)
Smoking, *n* (%) Current Never Past	17 (48.6%) 7 (20%) 11 (31.4%)	10 2 6	7 5 5
BMI, *n* (%) · Underweight · Healthy weight · Overweight · Obesity	1 (2.9%) 18 (51.4%) 11 (31.4%) 5 (14.3%)	0 11 5 2	1 7 6 3
DM2, *n* (%)	1 (2.9%)	1	0
Average SBP (mm Hg)	129.6 ± 7.6	131.6 ± 5.6	127.4 ± 8.8
Average DBP (mm Hg)	87.0 ± 5.0	86.2 ± 5.5	87.9 ± 4.4
Heart rate (bpm)	70.5 ± 11.46	70.5 ± 11.7	70.5 ± 11.46
Mean PPD (mm)	4.14 ± 0.49	4.15 ± 0.52	4.13 ± 0.47
Mean CAL (mm)	4.51 ± 0.71	4.64 ± 0.86	4.37 ± 0.49

Abbreviations; BMI, body mass index; bpm, beats per minute; CAL, clinical attachment loss; DBP, diastolic blood pressure; PPD, probing pocket depth; SBP, systolic blood pressure.

**Table 2:** Effects on blood pressure and periodontal changes ( *p* -value calculated between baseline and periodontal re-evaluation and between baseline and 6-month follow-up)

Parameters	Baseline	Periodontal re-evaluation 4–6 weeks		6-month follow-up	
SBP (mm Hg)	129.6 ± 7.6	124.9 ± 10.2	( *p* = 0.016) a	124.4 ± 9.8	( *p* = 0.007) a
DBP (mm Hg)	87.0 ± 5.0	83.6 ± 7.5	( *p* = 0.015) a	83.3 ± 6.1	( *p* = 0.003) a
PPD (mm)	4.14 ± 0.49	3.44 ± 0.52	( *p* < 0.001) a	3.41 ± 0.55	( *p* < 0.001) a
CAL (mm)	4.51 ± 0.71	3.78 ± 0.78	( *p* < 0.001) a	3.76 ± 0.81	( *p* < 0.001) a
BOP (%)	96.2	43.4	( *p* < 0.001) a	44.6	( *p* < 0.001) a

Abbreviations: BOP, bleeding on probing; CAL, clinical attachment loss; DBP, diastolic blood pressure; PPD, probing pocket depth; SBP, systolic blood pressure.

a Statistically significant.

In all visits, patients were asked if there had been any change of habits whether it was an improvement in dental hygiene or any lifestyle change such as smoking reduction, increase in physical activity, diet changes, or weight changes.

### Blood Pressure Measurements

Blood pressure measurements (SBP, DBP, and heart rate) were obtained by a trained operator, with a validated electronic upper-arm cuff (Boso Medicus Family 4 Bosch + Sohn GMBH U.CO. KG, Jungingen, Germany) according to the ESC guidelines. 2 Patients were asked not to talk during the measurements and not to exercise, smoke, or consume caffeine the previous 30 minutes to the appointment. When the patients arrived, measurements were taken after 5 minutes of rest and in both arms. If there were important differences between them, the arm with higher values was used. Measurements were taken three times at 2-minute intervals; the first one was discarded and the mean of the other two was used. The patients were with back support, feet flat on the floor, with the arm bare resting and with mid-arm at heart level. To perform the study and in order to include the patients, BP measurements were taken in the first two visits to the clinic. The baseline BP measurements presented are the average of the first- and the second-visit measurements.

### Periodontal Examination and Treatment

Periodontal treatment consisted of nonsurgical periodontal treatment (scaling and root planning), divided in two half-mouth sessions separated 7 to 10 days between them. Patients were locally anesthetized with articaine 4% epinephrine 1:100.000 (Septodont, Mataró, Barcelona, Spain) or mepivacaine 3% (Septodont) if necessary. Treatment was performed first with H3 ultrasound tip (Acteon Satelec, Acteon Médico-Dental Ibérica, Sentmenat, Barcelona, Spain) and then with Gracey curettes (Bontempi—American Eagle, Missoula, Montana, USA). Irrigation with oxygen peroxide was performed at the end of each session. Patients were given postoperative and dental hygiene instructions and were asked to rinse with chlorhexidine 0.12% + CPC 0.05% (Dentaid, Cerdanyola del Vallès, Barcelona, Spain) two times a day during 2 weeks following the treatment. Data was recorded by hand, checked for errors, and then converted to an electronic data sheet.

### Data Analysis

#### Variable Description

SBP and DBP levels were described as quantitative variables expressed in mm Hg. PPD and CAL were assessed at six points sites around each tooth, and were described as quantitative variables expressed in mm. For analysis purposes, the mean of all sites was calculated for each patient in each visit. BOP was described as a categorical variable (presence or absence of bleeding) and assessed for each tooth. For analysis purposes, the percentage of BOP was calculated for each patient in each visit. BMI was calculated as a quantitative variable but was classified in four groups: underweight ( *p* <18.5), healthy weight (18.5–24.9), overweight (25–29.9), and obesity (>30).

#### Statistical Analysis

Statistical analysis was performed with python scipy.stats. Shapiro–Wilk test was used to assess if variables were normally distributed. Paired samples Student's *t* -test was used to compare BP levels and PPD and CAL values before and after nonsurgical periodontal treatment. Normally distributed variables were reported as mean ± SD. Wilcoxon signed-rank test was used to compare BOP percentages before and after nonsurgical periodontal treatment. Relationship between the decrease of BP levels and the amount of periodontal improvement was assessed with Pearson or Spearman Correlation as needed. *p* -Value less than 0.05 was considered statistically significant.

## Results

**Fig. 1:**
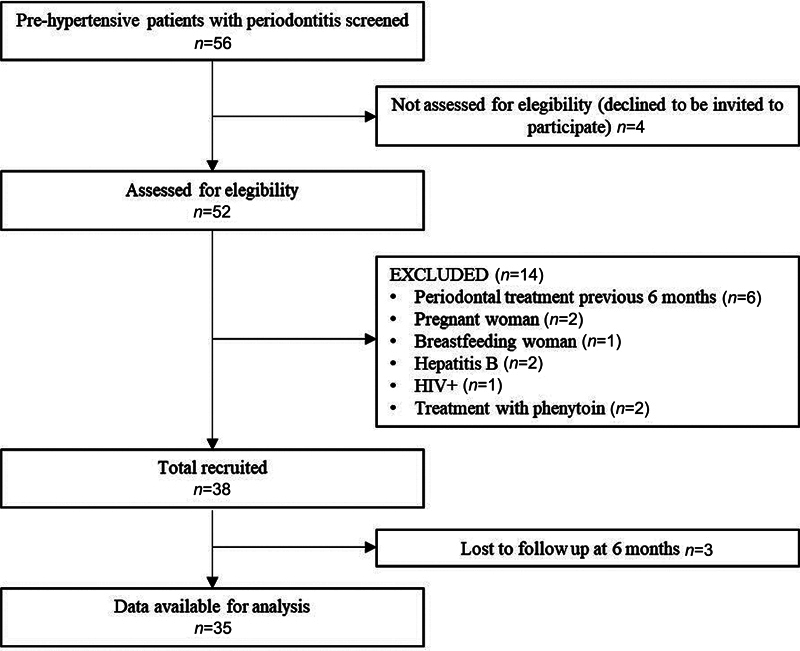
Flow diagram of patient inclusion and exclusion.

After a screening of 56 patients, 38 meet the inclusion criteria for adults with prehypertension and periodontal disease. Three patients did not reach the 6-month time point and were lost to follow-up. Finally, 35 patients (17 females and 18 males) finished the 6-month follow-up and were included in the study ( Fig. 1 ). Baseline characteristics of the patients included are shown in Table 1 .

### Effects of Periodontal Treatment on Blood Pressure

**Fig. 2:**
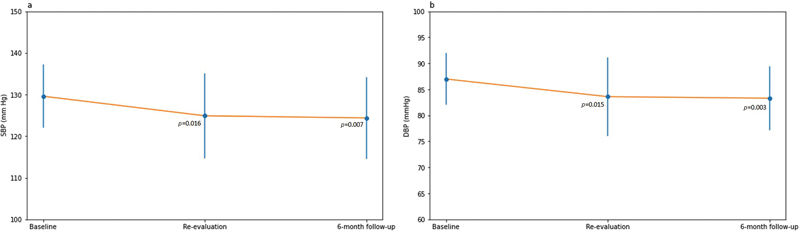
Systolic blood pressure (SBP) and diastolic blood pressure (DBP) during 6-month follow-up period. Parts **A** and **B** represent mean SBP and mean DBP, respectively. Vertical bars represent standard deviation (SD). *p* -Values were calculated with paired samples Student's t-test between baseline and re-evaluation visit and between baseline and 6-month follow-up visit.

There were statistically significant differences ( *p* < 0.05) of BP values before and after nonsurgical periodontal treatment ( Fig. 2 ).

**Fig. 3:**
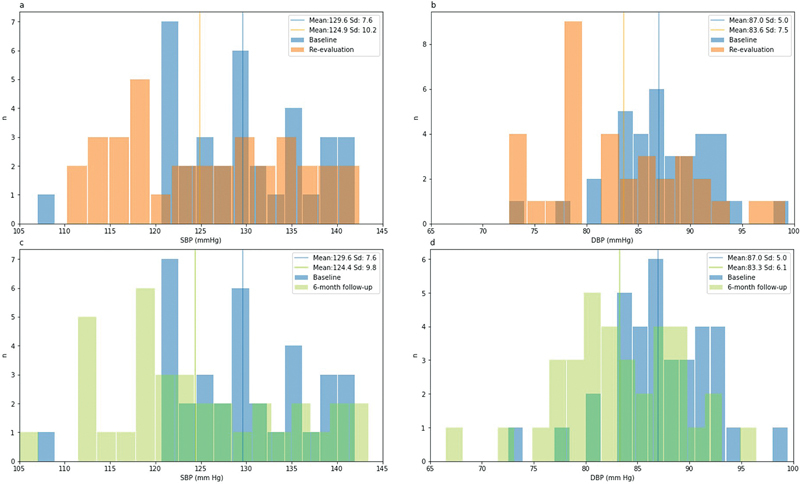
Histogram of blood pressure (BP) distribution. Parts **A** and **B** represent changes in systolic blood pressure (SBP) and diastolic blood pressure (DBP) distribution, respectively, between baseline and periodontal re-evaluation visit. Parts **C** and **D** represent changes in SBP and DBP distribution, respectively, between baseline and 6-month follow-up visit. Vertical lines represent BP mean values of each visit.

Periodontal treatment caused a significant reduction at the re-evaluation visit of SBP ( Fig. 3A ) and DBP ( Fig. 3B ) by 4.7 and 3.4 mm Hg, respectively. The effect was maintained over time and is reflected in the 6-month follow-up visit with a reduction in the SBP ( Fig. 3C ) and DBP ( Fig. 3D ) by 5.2 and 3.7 mm Hg, respectively.

### Effects of Periodontal Treatment on Periodontal Health

The dental treatment produced a substantial improvement in periodontal parameters of all participants when compared to baseline. The analyzed variables, PPD, CAL, and BOP, had statistically significant reduction during follow-up ( Table 2 ).

**Fig. 4:**
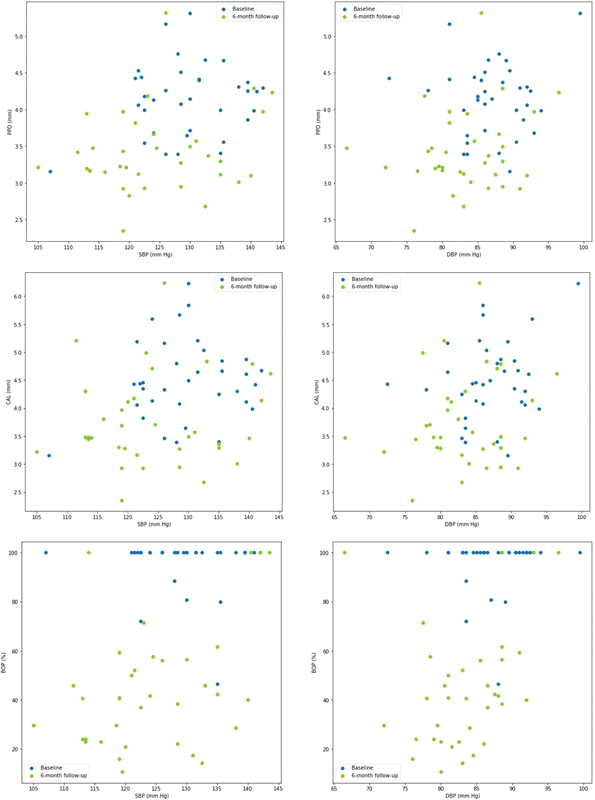
Relationship between periodontal parameters (probing pocket depth, PPD [mm], clinical attachment loss, CAL [mm], bleeding on probing, BOP [%]) and systolic blood pressure (SBP) and diastolic blood pressure (DBP; mm Hg) measurements (left and right column, respectively). Baseline values are presented in blue and 6-month follow-up values are presented in green.

There is a reduction in the mean values of PPD, CAL, and BOP percentages as well as the mean values of SBP and DBP between baseline and re-evaluation visit ( Supplementary Figure S1 , available in the online version) and between baseline and 6-month follow-up visit ( Fig. 4 ). However, there was no direct relationship between the amount of periodontal improvement and the amount BP levels improvement. No relevant changes in clinical data (toxic and healthy habits) were observed between visits. An important number of patients (82.8%) had an improvement of dental hygiene habits at 6 months follow-up ( Supplementary Table S1 , available in the online version). Age, sex, smoking habit, alcohol consumption, type of diet, physical activity, and changes in dental hygiene habits had no statistically significant impact in the improvement of BP levels.

## Discussion

Recent studies suggest a causal link between periodontitis and high BP 9 10 13 16 26 confirming that patients with severe periodontitis tend to have higher BP levels than patients with healthy periodontal tissues. Moreover, evidence suggests that periodontal treatment can improve BP levels. 18 19 20 21 22 23 27

This study found a statistically significant reduction in SBP and DBP both in the periodontal re-evaluation visit (4–6 weeks after the treatment) that was maintained at 6-month follow-up visit. Despite the heterogeneity in groups, follow-up protocols and types of study our findings are in line with recent studies regarding the effect of nonsurgical periodontal treatment in BP levels. BP reductions described in literature are highly variable from no decrease at all, 28 not statistically significant, 29 or reductions in 11.1 mm Hg. 9

In 2006 D'Aiuto et al 18 found reduction in SBP after periodontal treatment combined with antimicrobials of 7 ± 3 mm Hg at 2 months but this reduction was not stable at 6-month follow-up. Vidal et al in 2013 19 described a reduction both in SBP and DBP of 12.5 and 10.0 mm Hg, respectively, 6 months after nonsurgical periodontal treatment. In 2015, Hada et al 20 found a reduction in SBP of 7.1 mm Hg at 6 months. In 2016, Houcken et al 21 described a decrease in SBP of 2.9 mm Hg at 6 months. Similar results were found by Bizzarro et al 22 in 2017, which described a reduction of 2.7 mm Hg at 12 months in patients that received nonsurgical periodontal treatment which increased to 5.4 mm Hg reduction when treatment combined with antibiotics.

In 2017, Zhou et al 23 described a reduction in SBP and DBP of 12.57 and 9.65 mm Hg, respectively, at 6 months following scaling and root planning periodontal treatment. Similar results were published by Czesnikiewicz-Guzik et al 9 in 2019, which found a reduction in SBP of 11.1 mm Hg and DBP of 8.3 mm Hg 2 months after periodontal treatment. In this last case, BP measurements were taken with a 24-hour ambulatory blood pressure monitoring (ABPM). While this measurement method is more reliable in diagnosing high BP, some evidence suggests 11 that studies using ABPM show the greatest reduction both in SBP and DBP. Our findings are also consistent with recent large cross-sectional survey of 11,753 participants which showed that periodontal health is associated with better SBP profile by about 3 mm Hg and with lower odds of antihypertensive treatment failure. 30

The prevalence of high normal BP is estimated to be 30 to 50% of overall population 5 6 7 31 and is associated with three times more likelihood of developing hypertension in the future. 1 Therefore, any strategy focused on diagnosing and lowering BP levels on these patients is an important preventive public health goal. The study published by Kawabata et al 27 suggests that the presence of periodontitis can be a risk factor for developing hypertension in patients with prehypertension.

The exact mechanisms that link high BP and periodontitis remain unclear. Different factors are described in the literature. The most important pathomechanism seems to be systemic inflammation that may be exacerbated by local gingival inflammation, and secondary damage to the vascular endothelium. 11 Moreover, the bacteremia and oral pathogens dysbiosis that occur in periodontal diseases has an important role. Oral bacteria can influence nitric oxide production and this may produce metabolic abnormalities that contribute to BP levels rising. 11 Furthermore, recent evidence demonstrates common genetic predisposition factors for both diseases, 11 which can explain the frequent coexistence of them.

This study has not found a proportional relationship between the rate of improvement of periodontal values (PPD, CAL, BOP) and the rate of BP level improvement (SBP and DBP). This may be explained by the fact that performing nonsurgical periodontal treatment in patients with periodontitis decreases by itself gingival and systemic inflammation.

This simple periodontal treatment that patients need is already a benefit for the individual cardiovascular risk profile of patients. An improvement in only 5mm Hg in SBP can reduce stroke mortality by 14% and cardiovascular disease mortality by 9%. 26

Our clinical study has its limitations. The low number of patients included needs to be confirmed in a large cohort of prehypertensive patients. Moreover, there may be an overestimation of BP levels due to white coat hypertension. Twenty-four hours ABPM monitoring would be a very useful tool to confirm diagnostics. Also, a longer follow-up of 12 months would be needed to allow for therapeutic recommendations and conclusions. Finally, it should be considered that the decrease in BP may be due to the result of general healthy instructions and oral hygiene improvement apart from periodontal treatment.

Findings of this study suggest the importance and impact of periodontal treatment in BP levels in patients with prehypertension, and the importance of early detection of this subgroup of patients, who are usually underdiagnosed because they do not meet the strict criteria for hypertension.

Likewise, and just as important as the clinical findings, our study emphasizes the figure of the dentist in the context of public health. The role of professionals in dental clinics is fundamental in the primary and secondary prevention of arterial hypertension. The implementation of BP screening programs in patients with periodontitis and practical circuits where to refer affected patients is essential. Finally, the clinical practice of the dentist can be fundamental in the nonpharmacological control of this cardiovascular risk factor.

## Conclusion

This study shows that nonsurgical periodontal treatment can be effective in lowering BP levels in patients with prehypertension and periodontitis, without any antihypertensive medication. It also highlights the importance of dentists in prevention, detection, and control of high BP and cardiovascular risk
