# Supplementary material for: Impact of Nonsurgical Periodontal Treatment on Blood Pressure: A Prospective Cohort Study
Source: Eur J Dent. 2023 Sep 20;18(2):517–25. doi: 10.1055/s-0043-1772246 (PMC11132759; doi:10.1055/s-0043-1772246)
Supplement: Supplementary file 1 — Supplementary Material [file 10-1055-s-0043-1772246-s2322657.pdf]

Supplementary Table 1 Dental habits questionnaire at baseline and 6-month follow-up

**Baseline**	**Total ($n = 35$)**	**Male ($n = 18$)**	**Female ($n = 17$)**
Type of brushing	17	8	9
- Manual	13	7	5
- Electric	5	3	2
- Both			
Frequency of brushing	8	5	3
- 1/day	17	7	10
- 2/day	10	6	4
- 3/day			
Interproximal hygiene	25	12	13
- No	10	6	4
- Yes (1/day)	7	4	3
- Dental floss	3	2	1
- Interproximal brush			
Bleeding while brushing	2	0	2
- No	28	16	12
- Yes	5	2	3
- Occasionally			
Regularity dental appointments	11	7	4
- Yes	24	11	13
- No			
6-month follow-up	Total ($n = 35$)	Male ($n = 18$)	Female ($n = 17$)
Type of brushing	1	1	0
- Manual	25	12	13
- Electric	9	5	4
- Both			
Frequency of brushing	4	3	1
- 1/day	13	6	7
- 2/day	18	9	9
- 3/day			
Interproximal hygiene	2	1	1
- No	33	17	16
- Yes (1/day)	8	4	4
- Dental floss	25	13	12
- Interproximal brush			
Bleeding while brushing	26	12	14
- No	1	1	0
- Yes	8	5	3
- Occasionally			

 © 2023. The Author(s).

Supplementary Table 2 Clinical data at baseline

	Total ($n = 35$)	Male ($n = 18$)	Female ($n = 17$)
Cardiovascular risk factors and systemic diseases			
- Diabetes mellitus	1	1	0
- Dyslipidemia	1	1	0
- Hypothyroidism	2	0	2
- Asthma	2	1	1
Toxic habits			
- Smoking	17	10	7
- <10 cigarettes/day	6	3	3
- 10–20 cigarettes/day	9	5	4
- >20 cigarettes/day	2	2	0
- Alcohol consumption	5	1	4
- Never	29	16	13
- Occasionally	1	1	0
- Every day			
**Healthy habits**			
- Healthy/Mediterranean diet	32	16	16
- Regular carbonated/sugared drinks	5	4	1
- Physical activity	9	3	6
- Never	17	7	10
- Occasionally	6	5	1
- 2–3 times a week	3	3	0
- Every day			

 © 2023. The Author(s).

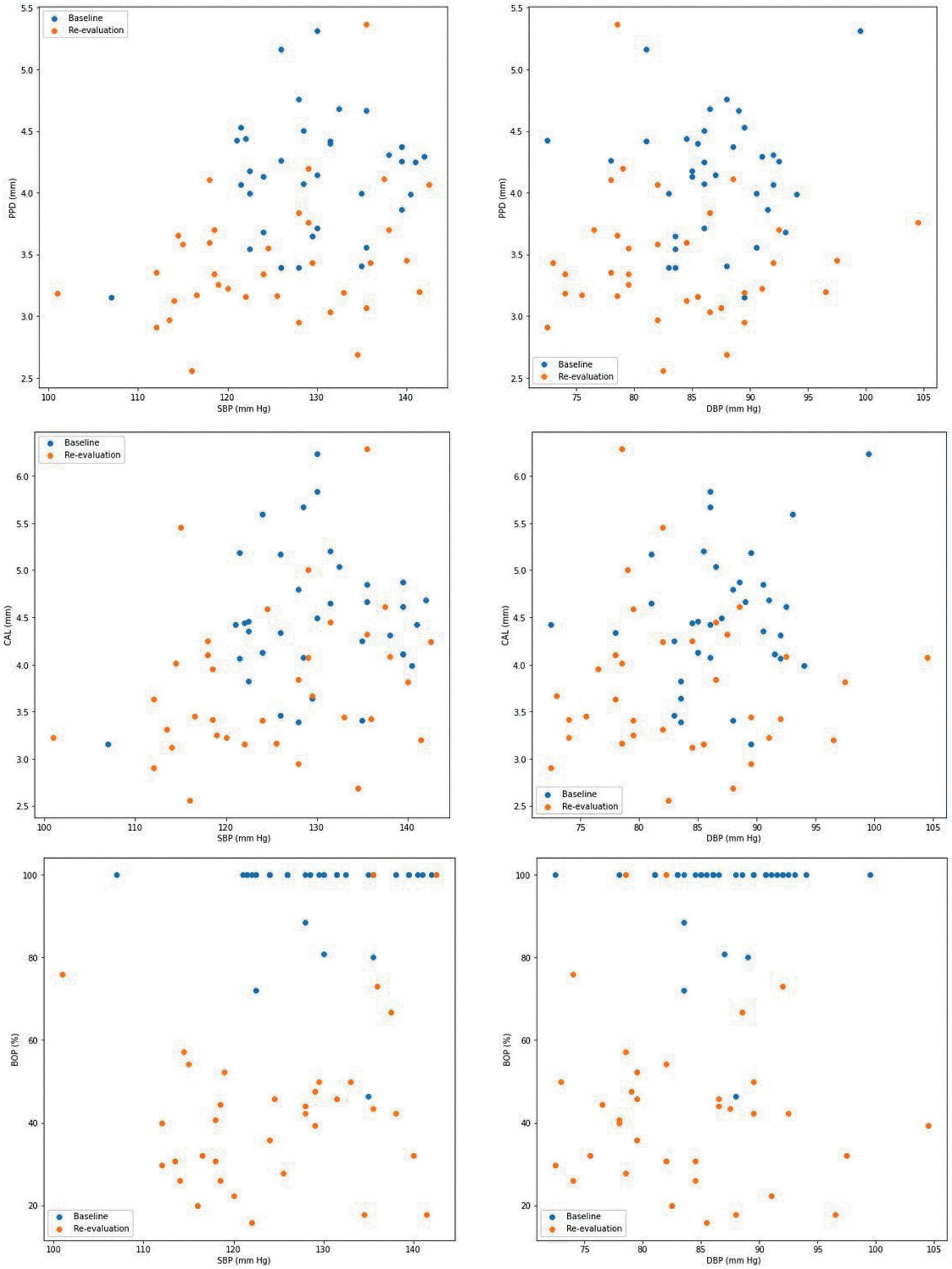

Supplementary Fig. 1 Relationship between periodontal parameters (probing pocket depth, PPD [mm], clinical attachment loss, CAL [mm], bleeding on probing [BOP]) and systolic blood pressure (SBP) and diastolic blood pressure (DBP; mm Hg) measurements. In blue baseline and in orange re-evaluation visit.

 © 2023. The Author(s).
